# The Photoinitiator Lithium Phenyl (2,4,6-Trimethylbenzoyl) Phosphinate with Exposure to 405 nm Light Is Cytotoxic to Mammalian Cells but Not Mutagenic in Bacterial Reverse Mutation Assays

**DOI:** 10.3390/polym12071489

**Published:** 2020-07-03

**Authors:** Alexander K. Nguyen, Peter L. Goering, Rosalie K. Elespuru, Srilekha Sarkar Das, Roger J. Narayan

**Affiliations:** 1 Joint UNC/NCSU Department of Biomedical Engineering, North Carolina State University, Raleigh, NC 27695, USA; aknguye2@ncsu.edu; 2 Office of Science and Engineering Laboratories, Center for Devices and Radiological Health, U.S. Food and Drug Administration, Silver Spring, MD 20993, USA; Peter.Goering@fda.hhs.gov (P.L.G.); Rosalie.Elespuru@fda.hhs.gov (R.K.E.); Srilekha.Das@fda.hhs.gov (S.S.D.)

**Keywords:** lithium phenyl (2,4,6-trimethylbenzoyl) phosphinate, gelatin methacryloyl, bioprinting, photoinitiator, light exposure, mutagenicity, cytotoxicity, photorheology

## Abstract

Lithium phenyl (2,4,6-trimethylbenzoyl) phosphinate (LAP) is a free radical photo-initiator used to initiate free radical chain polymerization upon light exposure, and is combined with gelatin methacryloyl (GelMA) to produce a photopolymer used in bioprinting. The free radicals produced under bioprinting conditions are potentially cytotoxic and mutagenic. Since these photo-generated free radicals are highly-reactive but short-lived, toxicity assessments should be conducted with light exposure. In this study, photorheology determined that 10 min exposure to 9.6 mW/cm^2^ 405 nm light from an LED light source fully crosslinked 10 wt % GelMA with >3.4 mmol/L LAP, conditions that were used for subsequent cytotoxicity and mutagenicity assessments. These conditions were cytotoxic to M-1 mouse kidney collecting duct cells, a cell type susceptible to lithium toxicity. Exposure to ≤17 mmol/L (0.5 wt %) LAP without light was not cytotoxic; however, concurrent exposure to ≥3.4 mmol/L LAP and light was cytotoxic. No condition of LAP and/or light exposure evaluated was mutagenic in bacterial reverse mutation assays using *S. typhimurium* strains TA98, TA100 and *E. coli* WP2 uvrA. These data indicate that the combination of LAP and free radicals generated from photo-excited LAP is cytotoxic, but mutagenicity was not observed in bacteria under typical bioprinting conditions.

## 1. Introduction

The growing waiting list for tissue and organ transplants is a critical and oft-cited reason for the importance of tissue engineering research. Three-dimensional bioprinting using photopolymers takes a front role, due to its ability to manufacture patient-specific device geometries. Photopolymerization provides several advantages in bioprinting, such as biocompatibility, high resolution lithography, and tunable viscoelastic properties [1,2,3]. Thus, extrusion bioprinting of a photopolymer containing cells is one popular method of creating tissue engineering constructs [4]. Despite the promise, photopolymer-based 3D bioprinting has not developed enough to find commercial success beyond in vitro models due, in part, to the complexity of the tissues and the large number of uncharacterized variables [5].

While different 3D printing apparatuses have unique considerations that affect outcomes, such as cell settling or high shear strains, the use of photopolymers during printing would expose cells to the photopolymer components and light characteristics used during crosslinking. Photopolymers are composed of a base polymer containing a reactive crosslinking moiety (e.g., acrylate, epoxide, and thiol-ene chemistries) and an appropriate photoinitiator compatible with this moiety (e.g., free radical generators, photoacid generators) [6]. Overall, acrylate and thiol-ene chemistries are the prevalent reactive moiety, both of which are used with free radical-generating photoinitiators; upon exposure to light, these types of photoinitiators generate free radicals to initiate the crosslinking reaction that can also be cytotoxic to encapsulated cells [7,8]. Many studies demonstrate low cytotoxicity related to their bioprinting process, but there is a dearth of reports that specifically focus on the toxic effects of light exposure in the presence of photopolymer [7]. Similar studies primarily focus on topical photosensitizers with existing in vitro methods, such as the KeratinoSens or hCLAT assays, both of which are being recognized by the Interagency Coordinating Committee on the Validation of Alternative Methods for human skin sensitizer screening [9,10,11]. However, systematic investigations focusing on light exposure and photoinitiator concentrations for the bioprinting field are absent.

Current studies reported in the literature are often demonstrations of a specific bioprinting apparatus or process; therefore, toxicity results are difficult to generalize to other systems. For example, Sabnis et al. investigated concurrent exposure of Irgacure 2959 photoinitiator and 365 nm light showing a cytotoxic dose-response correlated with only Irgacure 2959 concentration. The addition of 50 mg/L ascorbic acid, a radical scavenger, prevented cytotoxicity, which suggests that the mechanism of toxicity is related to free radical generation [12]. This study only investigated cell viability after exposing cells to photopolymer extracts, and did not assay encapsulated cells. Leonhardt et al. investigated the cytotoxicity and genotoxicity of photopolymer extracts showing minimal toxicities for the visible-light polymerized acrylate polymer [13]. The use of photopolymer extracts, as in these studies, is generally amenable to biocompatibility testing, but does not consider the initial burst of reactive oxygen species (ROS) generated when the photoinitiator is first exposed to light. Therefore, the use of extracts for biocompatibility testing of photopolymers may not accurately reflect the risks in bioprinting, where cells are exposed directly to photo-generated ROS. The photopolymer combination of gelatin methacryloyl (GelMA) and lithium phenyl (2,4,6-trimethylbenzoyl) phosphinate (LAP) is commercially available and widely cited for various biomedical applications, but there has been very little investigation into the mutagenicity potential of this material, especially considering the effect of light exposure during crosslinking. Given the literature demonstrating and proposing mechanisms of free-radical mediated mutagenicity, it is surprising that almost no studies have investigated the mutagenicity of the GelMA + LAP photopolymer, or of photoinitiators in general, despite the large number of studies evaluating this specific combination [14,15].

Kidney collecting duct cells are a well-known target of lithium, with kidney injury and disease being a common side effect of lithium treatment for psychiatric disorders [16,17,18,19]. The mechanism of lithium toxicity to collecting duct cells is related to the epithelial sodium channels (passive transport into the cell) having a greater affinity for lithium versus sodium and the sodium efflux ATPase pump having a lower affinity for lithium leading to accumulation within the cell [20,21]. Fibrosis is a late response to acute kidney injury, and is also observed after exposure to lithium chloride; thus, LAP, a lithium salt, may be more cytotoxic to collecting duct cells versus other cell types [22,23]. While alternative versions of this photoinitiator type exist, i.e., with a sodium instead of a lithium ion, with similar crosslinking efficiencies, changing the cation to sodium decreases solubility and increases cytotoxicity [24]. In this study, confluent M-1 mouse kidney collecting duct cells were considered, because these cells: (1) serve as a lithium target cell model, (2) exhibit epithelial polarity when cultured in well plates, and (3) form “domes” indicative of water transport [25]. LAP in cell culture medium is exposed to the in vitro equivalent of the apical membrane of collecting duct cells. Additionally, the use of this cell model would expand the scope to technologies that could directly contact native tissue, such as tissue adhesives, injectable wound dressings, or hand-held extrusion printers for bioprinting during surgery [26,27,28]. Therefore, the objectives of this study were to: (1) characterize light exposure conditions typical of bioprinters, using LEDs as a light source; (2) assess whether LAP with concurrent light exposure enhances cytotoxicity in confluent M-1 cells; and (3) determine if exaggerated exposure conditions associated with high cytotoxicity are mutagenic using bacterial reverse mutation assays.

## 2. Materials and Methods

### 2.1. Light Source Characterization and Photorheology

Two different LED light sources with a 405 nm nominal peak emission wavelength were evaluated for their emission spectra and irradiance using a HR4000 spectrometer (Ocean Insight, Largo, FL, USA) and an Ophir Vega with a PD-300UV photodiode detector (Ophir, Jerusalem, Israel), respectively. To characterize the beam profile of a M405LP1 LED (Thorlabs, Newton, NJ, USA) used for photorheology, the photodiode was masked with an approximately 1-mm pinhole and moved through the center of the beam; the LED chip was placed 150 mm vertically from the detector. Relative power was measured every 1 mm through the center of the beam. To illuminate the larger area required for the mammalian and bacterial assays, a higher power 405 nm LED array (Amazon, Seattle, WA, USA) was used. The array was embedded into the bottom of a polystyrene box and oriented upward. A 55 × 70 mm^2^ cutout on top of the box allowed light to illuminate well plates from the bottom.

Photorheology measurements of 10 wt % GelMA (Cellink, Boston, MA, USA) formulations were made using an MCR302 rheometer (Anton Paar, Ashland, VA, USA) with a fused silica lower plate and a 20-mm parallel plate geometry. GelMA used in this study was derived from 300 bloom porcine gelatin with a 54% degree of methacrylation, and was sterile filtered through a 0.22 µm polyethersulfone membrane by the manufacturer before lyophilization. The temperature was maintained at 37 °C, and the gap distance was set at 0.5 mm; this distance was varied during the experiment to maintain a constant 0.0 N normal force. The M405LP1 LED was set to 3.0 mW/cm^2^. The linear viscoelastic region (LVER) was determined with a pair of pilot tests on 10 wt % GelMA containing 17 mmol/L LAP exposed to >10 min of light before measurement; a frequency sweep from 0.2–20 Hz at a constant 1% shear strain, followed by a shear strain sweep between 0.01% and 1000%, were performed. The photorheology experiments were conducted with a 60 s pre-shear at 5% shear strain, followed by triggering of the light source and simultaneous oscillatory measurement at 5% strain and 5 Hz for 15 min. An additional run investigated the crosslinking kinetics of 10% GelMA with 3.4 mmol/L LAP, after a nitrogen purge through the solution for ~2 min.

### 2.2. Cytotoxicity Assays

M-1 mouse collecting duct cells (ATCC, Manassas, VA, USA) were incubated (37 °C, 5% CO_2_, 95% RH) in DMEM:F12 medium with 5 vol % FBS and 5 µmol/L dexamethasone (Sigma Aldrich, St. Louis, MO, USA) for 10 days in 96-well plates with media changes every 2 or 3 days. Cell layers cultured for at least 10 days were observed to form domes indicative of ion transport; this observation is qualitative but is typical of healthy confluent layers [25].

Alamar Blue (AB) and Neutral Red Uptake (NRU) cytotoxicity assays were conducted on M-1 collecting duct cells exposed to LAP (Sigma Aldrich, St. Louis, MO, USA), with or without light exposure. The first set of assays without light exposure was performed by exposing cell layers to 3.4, 5.1, 6.8, 17, 25.5, 34, 51, or 68 mmol/L LAP or lithium chloride (LiCl) (Sigma Aldrich, St. Louis, MO, USA) in cell culture medium for 24 h, before conducting the AB and NRU assays. The second set of experiments with light exposure was conducted by adding 0, 3.4, 5.1, 6.8, 17 mmol/L of either agent to cell culture medium, followed immediately by exposure to 10 min of 9.6 mW/cm^2^ 405 nm light from the LED array. Both sets of plates were incubated for 24 h, before the AB and NRU assays were performed. In both sets of experiments, cell layers exposed to cell culture medium, but not exposed to light, or cell culture medium containing 1 mmol/L cadmium chloride, were used as the negative and positive cytotoxicity controls, respectively.

For the AB assay, contents of all wells were aspirated and replaced with cell culture medium containing 10 vol % AB dye (ThermoFisher, Waltham, MA, USA). Plates were incubated for 2 h before fluorescence measurements were taken on a Tecan M1000 at 560 nm excitation and 585 nm emission wavelengths.

For the NRU assay, a 4 mg/mL Neutral Red (Sigma Aldrich, St. Louis, MO, USA) stock solution in PBS was diluted to 40 µg/mL in cell culture medium and placed at 37 °C overnight prior to assay. Neutral Red solutions were centrifuged at 600 g for 10 min the day of the assay, and the supernatant was retained to produce the NRU working solution. NRU desorb solution was produced by mixing 12.5 mL of ultrapure water (Millipore, Burlington, MA, USA), 12.5 mL of 200-proof ethanol (Decon Labs, King of Prussia, PA, USA), and 250 µL glacial acetic acid (Acros Organics, Fair Lawn, NJ, USA). The contents of all wells were aspirated, the cell layers rinsed with 150 µL PBS, then 100 µL of NRU working solution added. After 2 h incubation, plates were inspected under a phase contrast microscope to check for unintended crystallization of the dye. The cell layers were rinsed 2× with 150 µL PBS, then 150 µL of the NRU desorb solution was added. The plates were agitated for >10 min on a shaker, before fluorescent reading at 534 nm excitation and 616 nm emission wavelengths.

Within each replicate experiment, the signal from 4 or 5 identical wells for each toxicant concentration were averaged. Results are reported as the means and standard deviations of N = 3 independent replicate experiments. Statistically significant differences (*p* < 0.05) were determined by multiple two-tailed t-tests (Graphpad Prism 6, Graphpad Software, San Diego, CA, USA).

### 2.3. Ames Bacterial Reverse Mutation Assay

The Ames bacterial assay used in this study consists of exposing the bacteria to both the potential mutagen and light in a 96-well plate and plating on minimal glucose plates, using an overlay method [29]. Minimal glucose agar was composed of 16 g/L agar (Difco, Detroit, MI, USA), 20 mL/L of 50× VBE salt (Moltox, Boone NC, USA), and 21.5 g/L glucose (Sigma Aldrich, St. Louis, MO, USA) in DI water; VBE salts and previously autoclaved 40 % *w/v* glucose were added to the autoclaved agar solution once cooled to approximately 50–60 °C, then poured in volumes of ~30 mL each into 100 mm polystyrene petri dishes and allowed to solidify. Plates were stored at 4–8 °C until use. Top agar was composed of 6 g/L agar, and 5 g/L NaCl (Sigma Aldrich, St. Louis, MO, USA) which was autoclaved; histidine/biotin (His/Bio) or tryptophan (Trp) was added to a final concentration of 4.6 mg/L, once the solution cooled to approximately 50–60 °C. Aliquots of top agar containing His/Bio or Trp were stored at room temperature until use. Before each replicate experiment, bottom agar plates were placed in an incubator at 37 °C, and the top agar melted then cooled to 45 °C on a heating block. Stock solutions of strain-specific positive control compounds, 500 µg/mL 4-nitroquinoline-1-oxide (4-NQO) (Sigma Aldrich, St. Louis, MO, USA) for *E. coli* or 200 µg/mL 2-nitrofluorene (2-NF) (Sigma Aldrich, St. Louis, MO, USA) for *S. typhimurium*, were made in dimethylsulfoxide and stored frozen until use.

*S. typhimurium* (strains TA98 and TA100) and *E. coli* (strain WP2 uvrA) (Moltox, Boone NC, USA) from stocks frozen at −70 °C were cultured overnight in Oxoid, or Luria broth (ThermoFisher Scientific, Waltham, MA, USA), respectively, with 12 h agitation at 37 °C in fluted flasks on a shakeplate. Prior to any replicate experiment, optical density measurements at 600 nm (OD_600_) of the overnight cultures were taken with a Genesys 20 spectrophotometer (ThermoFisher Scientific, Waltham, MA, USA); all overnight cultures had an OD_600_ of approximately 1 prior to use in each assay. A 2× bacteria suspension was made by centrifuging 4 mL of the overnight culture at 8500 g for 30 a, and resuspending the pellet in 2 mL of PBS.

On 48-well plates, one well for each treatment and control condition was designated: well (1) PBS (negative control) alone; well (2) 0.5 µg/plate 4-NQO or 0.4 µg/plate 2-NF; well (3) 18.5 mJ/cm^2^ UV-C exposure, (4) LAP 34 mmol/L alone; and wells (5–10) LAP (0, 3.4, 8.5, 17, 25.5, and 34 mmol/L) + 10 min 9.6 mW/cm^2^ 405 nm light. Each well received 150 µL of 2x bacteria suspension and 150 µL of a solution containing PBS, positive control compound, or 102 mmol/L LAP, to achieve the appropriate concentrations. A separate plate containing the bacteria and PBS for UV-C treatment was exposed to 3 s of UV-C radiation in a UV crosslinker (Spectronics Corporation, New Cassel, NY, USA) corresponding to a radiant exposure of 18.5 mJ/cm^2^. The plate containing the remaining treatment groups were exposed to 10 min 9.6 mW/cm^2^ 405 nm light. Immediately after light exposure, 150 µL of the appropriate nutrient broth was added to all wells and the plates incubated for 30 min at 37 °C for mutant expression before plating.

Within each replicate, duplicate plates were produced from each well with each plate made by adding 150 µL of the well contents to 2 mL of top agar prewarmed to 45 °C, followed by immediate plating on bottom agar plates prewarmed to 37 °C. Plates were flipped once solid and incubated for 2 days before counting colonies on a SphereFlash automated colony counter (IUL Micro, Barcelona, Spain). Results are reported as the means and standard deviations of N = 3 or 2 independent replicate experiments.

## 3. Results

### 3.1. Light Source Characterization and Photorheology

Since achieving even illumination of all samples was a major concern, a LED light source and an in-house light box containing a consumer LED array were characterized for their irradiance over the sample area and emission spectra. The Thorlabs M405LP1 and consumer LED array had a narrow, symmetrical emission spectrum with peak emission of 409 ± 7 nm full width half maximum (FWHM) and 406 ± 7 nm FWHM respectively. The M405LP1 exhibited peak irradiance in the center of the beam and decreased to 99% of peak values 10 mm from the center when the LED chip is 150 mm above the detector. Irradiance over the entire 20 mm diameter parallel plate geometry would be within 1% of the set value. Irradiance at the sample platform over the consumer LED array had unpredictable hotspots due to the array and multiple diffuse reflections inside the box, but maximum and minimum irradiance readings were 9.8 and 9.4 mW/cm^2^ over the entire 55 × 70 mm^2^ area, with no ability to alter the intensity of the light source after lightbox assembly. Irradiance over any sample in the biological assays should deviate within 2% of 9.6 mW/cm^2^.

The LVER of fully crosslinked 10% GelMA was determined first with a frequency sweep from 0.2–20 Hz at a constant 1% shear strain. The approximately 5500 Pa storage modulus value was found to be on par with the final storage modulus determined in the main crosslinking experiment, and was indicative of a fully crosslinked gel. A subsequent sweep between 0.01% and 1000% strain at 5 Hz revealed a drop in the storage modulus starting at approximately 10% shear strain. Therefore, 5% shear strain at 5 Hz was determined to be within the LVER of 10% GelMA and was used for the main photorheology experiment to track the rate of crosslinking.

**Figure 1:**
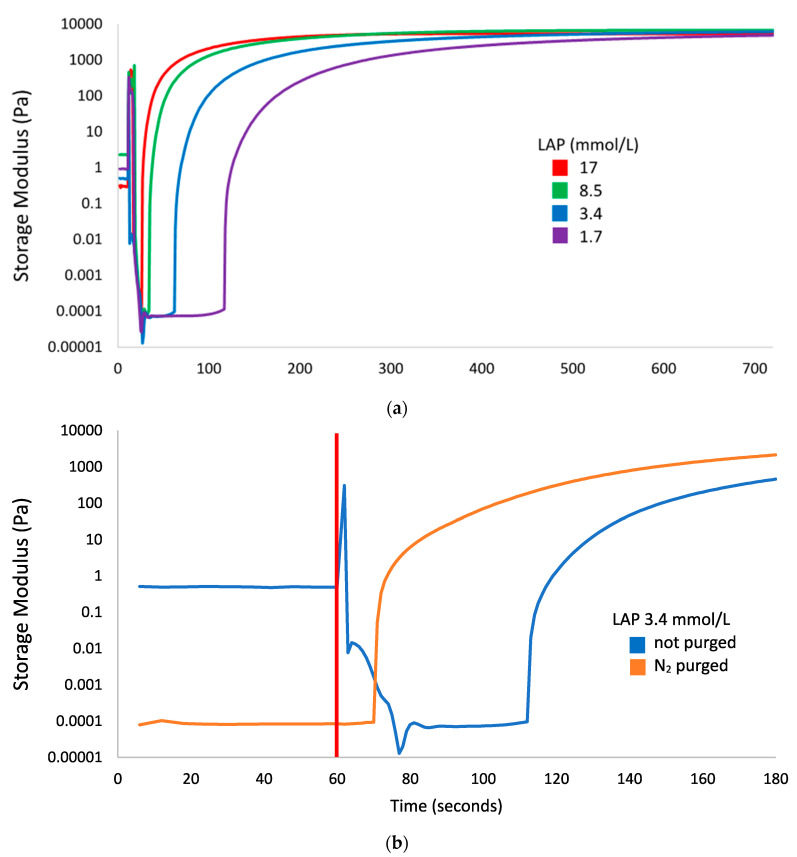
Storage modulus of 10% gelatin methacryloyl (GelMA) with lithium phenyl (2,4,6-trimethylbenzoyl) phosphinate (LAP) photoinitiator tracked by photorheology; the 405 nm light source (3 mW/cm^2^) was switched on at 60 s for all measurements. (**a**) Crosslinking of GelMA with 17 (red), 8.5 (green), 3.4 (blue), and 1.7 (purple) mmol/L LAP. (**b**) Crosslinking of non-nitrogen-purged (blue) and nitrogen-purged (orange) GelMA with 3.4 mmol/L LAP, with a focus on the time between light exposure and a rise in storage modulus; the red line denotes the start of light exposure.

Figure 1 summarizes four features of the photorheology results: (1) lag time between start of light exposure and increase of the storage modulus, (2) the final storage modulus after excess light exposure, (3) the rate of increase of the storage modulus, and (4) an artifact starting immediately after triggering the light source. This artifact is attributed to a software error where shear strain readings reached between 700% and 1700%, and torque readings reached extremely high levels (up to 0.03 N∙m) following an invalid data point. This caused the storage modulus calculations to be artificially high, but these readings stabilized to baseline levels within 13 s. This artifact was not present on the nitrogen purged sample that was collected after an equipment reset, and did not have the invalid data point.

The lag time is inversely proportional to the LAP concentration with gels containing 17, 8.4. 3.4, and 1.7 mmol/L LAP taking 17, 25, 53, and 108 s after initiating light exposure and before any marked increase of storage modulus. This lag time is greatly reduced after nitrogen purging; comparing the storage modulus curves of GelMA + 3.4 mmol/L LAP with and without nitrogen purging in Figure 1b, the lag time was shortened from 53 to 10 s. The final storage modulus of all gels approached the same value at approximately 6000 Pa. Times to reach >95% of the final storage modulus for each gel were 319, 473, 720, or 995 s for 10% GelMA with 17, 8.4. 3.4, or 1.7 mmol/L LAP exposed to 3.0 mW/cm^2^ 405 nm light, respectively. The light exposure condition of 600 s of 9.6 mW/cm^2^ light used in the cytotoxicity and Ames assays would be sufficient to fully crosslink GelMA containing ≥3.4 mmol/L LAP, and would be a representative light exposure scenario for cells undergoing bioprinting using an LED light source.

### 3.2. Cytotoxicity Assays

**Figure 2:**
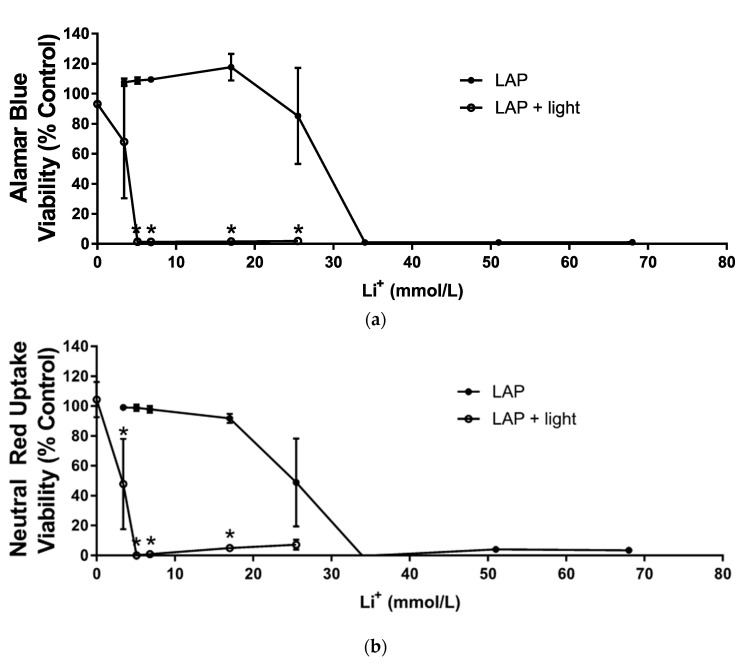
Viability of mouse M-1 collecting duct cell monolayers exposed to varying concentrations of LAP (3.4, 5.1, 6.8, 17, 25.5, 34, or 68 mmol/L) and not-exposed (•) or exposed to 9.6 mW/cm^2^ 405 nm light (o). LAP concentrations are represented as equivalent Li concentrations. Results are normalized to the viability of monolayers exposed only to PBS and not exposed to light. CdCl_2_ (1 mmol/L) was used as the positive control; viability averaged 0% for both assays (data not shown). (**a**) Viability determined by the Alamar Blue assay. (**b**) Viability determined by the Neutral Red Uptake assay. Values represent means ± standard deviations of N = 3 independent replicate experiments. Values that appear to be missing error bars are cases where the standard deviation is smaller than the symbol size. Asterisks (*) denote treatments where the viability of monolayers exposed to LAP is significantly different (*p* < 0.05) from the viability of monolayers exposed to LAP and light.

Confluent M-1 collecting duct cell layers were exposed to various concentrations of LAP or LiCl with or without 9.6 mW/cm^2^ 405 nm light exposure and the cell viability assessed with both the AB and NRU assays (Figure 2). The dose range for the cytotoxicity assays without light were centered around 17 mmol/L (0.5 wt % LAP), which is a common LAP concentration used in bioprinting. Cytotoxicity of LiCl was measured in parallel.

Exposure to LiCl with or without light slightly reduced viability. Cells exposed to the maximum investigated LiCl concentration (68 mmol/L) reduced viability down to 80% and 94% viability measured by the AB and NRU assays, respectively (data not shown). No differences in viability were observed between the treatments with LiCl alone and LiCl plus light exposure (data not shown).

Generally, the viability, as reported in both assays for the LAP dose response curve, was extremely sensitive, with concentrations yielding near 100% or near 0% viability flanking a single inflection point. Between 3.4 and 17 mmol/L LAP without exposure to light, viability in the AB assay was above 100% viability, and had an increasing trend over this concentration range, while viability in the NRU assay remained below 100% and had a decreasing trend. AB and NRU assays both reported a sharp drop in viability at 25.5 mmol/L LAP; at higher concentrations, all cells were dead as viability was essentially zero %.

Cells exposed to light alone (no LAP) exhibited no change in viability compared to controls. Cell viability at 3.4 mmol/L LAP, the lowest investigated dose, with concomitant light exposure diminished to 68% and 48% for the AB and NRU assays, respectively. Only the result of the NRU assay was significant at this point, however, the results show that the EC_50_ (effective concentration that kills 50% of the cells) for both assays was approximately 10-fold more potent for LAP + light, compared to LAP alone. Both assays showed that for cells exposed to LAP concentrations higher than 3.4 mmol/L LAP with concomitant exposure to light, viability was near zero.

### 3.3. Ames Bacterial Reverse Mutagenicity Assay

**Figure 3:**
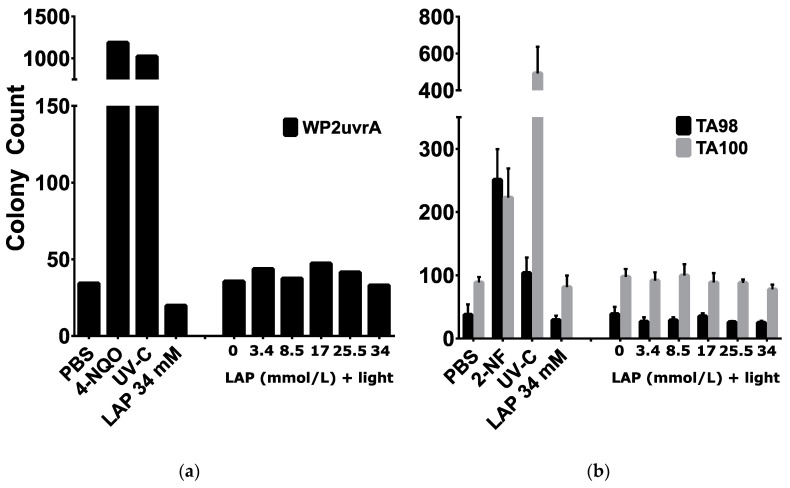
Mutagenicity assessment using bacterial reverse mutation assays. Reverse mutant colony counts of *E. coli* (WP2uvrA, (**a**)) and *S. typhimurium* (TA98 or TA100, (**b**)) exposed to PBS (negative control), 0.5 µg/plate 4-NQO or 0.4 µg/plate 2-NF (strain-specific positive controls), 18.5 mJ/cm^2^ UV-C light, 34 mmol/L LAP, or various LAP concentrations with exposure to 10 min of 9.6 mW/cm^2^ 405 nm light. For *E. coli* (**a**), values represent the average of N = 2 independent replicate experiments. For *S. typhimurium* strains (**b**), values represent means ± standard deviations of N = 3 independent replicate experiments.

Bacterial reverse mutagenicity assay results are summarized in Figure 3. All plates had an intact bacterial lawn, which indicated the lack of overt cytotoxicity; colonies present on the plates are attributed to reverse mutation. Baseline reverse mutation rates of WP2uvrA, TA98, and TA100 were 34, 38, and 89 revertant colonies, respectively. Strain-specific positive controls (i.e., 4-NQO for WP2uvrA and 2-NF for TA98 and TA100) resulted in a marked increase in colony counts. For all strains, either the control chemical mutagen, UV-C positive control, or both resulted in a positive mutagenicity assessment. In contrast, all treatments with LAP with or without light in all strains tested resulted in no apparent mutagenicity; colony counts were on par with the negative control.

## 4. Discussion

GelMA is made photosensitive with addition of LAP photoinitiator and is crosslinked with exposure to 405 nm light. Crosslinked GelMA is solid at cell culture temperatures (37 °C) and has a shear elastic modulus proportional to its concentration when fully crosslinked. Both LAP concentration and 405 nm light intensity are positively correlated with crosslinking rate, but do not affect final elastic modulus [30]. Photo-crosslinking has been traditionally performed with curing lamps, such as mercury or xenon arc lamps with filtered outputs for their high output power, but the high heat generation, high power draw, large volume, and relatively high cost of these lamps have pushed for the use of LEDs in portable light sources, such as dental curing lights and commercially-available bioprinters [28,31,32]. The use of 405 nm LEDs in this study is typical of bioprinters using LAP for these reasons. The use of 3 mW/cm^2^ irradiance in the photorheology studies was mainly limited by the desire to achieve even illumination over the entire 20 mm geometry. Most of the LED power is discarded to result in only a small emission angle to the sample; increasing the irradiance by moving the LED closer or by including focusing optics is associated with decreasing the evenness of the irradiance field. Since the polymerization rate is proportional to the square root of light intensity, the use of this lower irradiance can justify exposure conditions using the 9.6 mW/cm^2^ irradiance used in the biological assays [30].

Photorheology results were evaluated mainly for the light exposure time required to reach 95% of the maximum achieved storage modulus for each LAP concentration investigated. However, the lag time between the start of light exposure and the beginning of polymerization is an important artifact. This lag time is inversely proportional to the photoinitiator concentration, and is attributed to radical scavenging by oxygen [33]. To test this hypothesis, nitrogen purging of the 3.4 mmol/L LAP gel reduced the lag time, compared to the 17 mmol/L LAP gel. While nitrogen purging is a common method to improve the polymerization performance of acrylate photopolymers, this process is not compatible with cell-laden gels used in bioprinting [34]. Nevertheless, the result that the 3.4 mmol/L LAP gel required 720 s to fully polymerize justifies the exaggerated exposure from the higher 9.6 mW/cm^2^ irradiance for 600 s used in the subsequent studies.

Since studies have shown that both photoinitiator concentration and light exposure are associated with toxic effects, conventional wisdom states that these parameters should be carefully controlled [12,35]. For example, underexposure would result in imprecise control of the mechanical properties and an increase in the concentration of unreacted monomer, while overexposure would expose cells to additional phototoxic effects while not significantly affecting the mechanical properties. Multiple studies report photopolymer tissue engineering constructs with low cytotoxicity, and/or the ability of cells to proliferate within the construct after printing [28,36,37]. The success of this technique is contrary to the assumption that free-radical generating photoinitiators produce toxic effects, including genotoxicity, from reactive oxygen species [14,38]. Therefore, the second and third objectives of this study were to determine whether concomitant exposures to LAP and light under conditions typical of bioprinting were cytotoxic and/or mutagenic.

Cytotoxicity assays were performed using confluent mouse kidney M-1 collecting duct cell layers, which exhibited evidence of active water transport, i.e., “domes”, attributed to water transport between the cell layer and wellplate bottom [25]. Lithium ion is a known nephrotoxicant, whose mechanism of toxicity is related to the inhibition of AQP-2 transport to the cell membrane, thereby causing diabetes insipidus [39]. However, diabetes insipidus isn’t the major concern in this study versus lithium accumulation within the kidney, risk of renal fibrosis, and damage to various organelles; karyolysis and cell fragmentation were observed in the kidneys of rats exposed to lithium chloride [21,23,40]. Results in both the Alamar Blue and Neutral Red Uptake assays agree that lithium ion concentrations typically associated with LAP can be mildly cytotoxic; However, 95% viability from 17 mmol/L LiCl exposure (i.e., equivalent to 0.5 wt % LAP) determined with the NRU assay is a generally accepted viability for cells grown in vitro; this effect should be less pronounced in other cell models not as sensitive to lithium. At LAP concentrations and exposure times typically used in bioprinting, lithium ions were not identified as a significant cytotoxic factor.

In contrast, LAP exposure to M-1 collecting duct layers in the absence of light was cytotoxic at 17 mmol/L corresponding to 0.5 wt % LAP, a commonly used concentration in bioprinting. The exposure duration was 24 h, which represents an exaggerated exposure compared to bioprinted constructs, where LAP would be diluted by cell culture medium after crosslinking. With exposure to light, an immediate reduction in viability was observed at the lowest LAP concentration corresponding to 0.1 wt % LAP. This result is corroborated in a study by Duchi et al. where sheep adiopose derived stem cells exhibited around 20% viability after exposure to 0.1% LAP, albeit with 700 mW/cm^2^ 365 nm light for 10 s [28]. Bioprinting with similar LAP and light conditions generally does not elicit this cytotoxic response, so this discrepancy is attributed to a protective effect of GelMA and cell media used during bioprinting. For example, the presence of antioxidants, such as N-acetylcysteine, was shown to be protective against oxidative damage [41]. This strategy was also used to develop a photoprotective supplement composed of antioxidants, which prevented light-induced cytotoxicity in neuronal culture exposed to 360 kJ/m^2^ 470 nm light [42]. This protective effect was also observed in bone marrow stromal cells encapsulated with methacrylated hyaluronic acid or polyglycerol; neither Irgacure 2959 concentration or radiant exposure from 6 mW/cm^2^ 365 nm light for 5 min caused any statistically significant differences in cell viability or proliferation versus the non-irradiated control [43]. In the absence of the encapsulating polymer, increasing light exposure and/or Irgacure 2959 concentration caused drops in viability and proliferation [44]. High viability (~96%) was also observed in human neonate foreskin fibroblasts embedded in polyethylene glycol diacrylate with 2.2 mmol/L LAP exposed to 5–10 min of 10 mW/cm^2^ 405 nm light [30]. In the present study, in vitro assays performed in the absence of GelMA likely exaggerated cytotoxicity typically observed when using this material in bioprinting. All investigated LAP concentrations with exposure to light were highly cytotoxic to the M-1 collecting duct cells compared to LAP alone, and this confirms the hypothesis that concurrent LAP and light exposure is more cytotoxic than LAP exposure alone.

A modified Ames bacterial mutagenicity assay was conducted with LAP and light exposures that were cytotoxic to M-1 cells, to determine whether these exposures are mutagenic. This modification involves light exposure to the bacteria and toxicant prior to incubation, and has been previously used to determine mutagenicity from concomitant light and exposure to sunscreen and cosmetic [44,45] compounds. The results in the present study show that the positive and negative controls performed adequately within defined parameters, resulting in valid assays; however, mutagenicity assessment for LAP concentrations ranging from 0 to 34 mmol/L with or without 10 min of 9.6 mW/cm^2^ 405 nm light exposure were negative. OECD TG471 guidelines recommend a battery of four *S. typhimurium* strains (including TA98 and TA100) and additional choice of strain (including WP2 uvrA) which detects “oxidizing mutagens, cross-linking agents and hydrazines” [29]. While this study only used two *S. typhimurium* strains and one *E. coli* strain, recent studies showed that using only TA98 and TA100 detected 93% of the >10000 compound database [46]. Since ROS is expected to be an oxidizing mutagen, the TA100, TA98, and WP2 uvrA test battery used in this assay should have a high chance of detecting mutagenicity. Thus, the negative result observed is surprising. Although the addition of S9 metabolic activation is a customary procedure for testing chemicals that might require metabolic activation for genotoxicity, S9 was not deemed relevant to this study, because we are exploring another mode of activation: light-based photo-activation. The Ames assay is widely-used and is predictive of 70–90% of carcinogens, but the use of the Ames assay alone for a negative mutagenicity assessment is insufficient as a definitive stand-alone result [47,48]. The reasons for negative results include inadequate dosing (e.g., failure of uptake), failure to detect mammalian-specific mutagens, or chemical-specific requirements not met, in addition to a true lack of mutagenicity. Further investigations are needed to define the mutagenic status of LAP plus light, especially given the cytotoxicity observed.

Despite being a popular, well-cited, and commercially-available photoinitiator for use with bioprinting, there are no reports of studies evaluating the mutagenicity of LAP to the authors’ knowledge. However, an EU report on the chemically similar trimethylbenzoyl diphenyl phosphene oxide (TPO), which is used primarily in photo-crosslinkable nail polish, finds that TPO is not mutagenic based on data from multiple in vitro and in vivo assays; however, no study took light exposure into account [49]. TPO was also investigated alongside camphorquinone, in combination with various acrylate-based polymers, and found to be marginally genotoxic determined with the Comet assay, although light exposure was not assessed [50]. Based on these studies, LAP was not expected to be mutagenic in the absence of light exposure, but our results thus far do not support our hypothesis that LAP with light exposure would be mutagenic, given the expected generation of reactive oxygen species and the toxicity observed in mammalian cells.

## 5. Conclusions

The cytotoxicity of LAP to confluent M-1 collecting duct cells was significantly enhanced with exposure to 405 nm light with increased cytotoxicity observed at concomitant exposures of >3.4 mmol/L LAP (0.1 wt % LAP) and 10 min of 9.6 mW/cm^2^ light, conditions occurring during LAP use as a photoinitiator. However, LAP concentrations up to 34 mmol/L (1.0 wt %), an atypically high concentration for bioprinting, were not mutagenic in bacteria, even after exposure to light. This study supports that use of LAP photoinitiator and free radicals generated from photo-excited LAP can be cytotoxic to cells undergoing bioprinting. Further studies are needed to more definitively demonstrate the lack of mutagenicity of LAP and light exposure conditions typical in bioprinting.

## References

[1] Stampfl J., Baudis S., Heller C., Liska R., Neumeister A., Kling R., Ostendorf A., Spitzbart M. (2008). Photopolymers with tunable mechanical properties processed by laser-based high-resolution stereolithography. J. Micromech. Microeng..

[2] Larsen E.K.U., Larsen N.B., Almdal K., Larsen E.K.U., Larsen N.B., Almdal K. (2016). Multimaterial hydrogel with widely tunable elasticity by selective photopolymerization of PEG diacrylate and epoxy monomers. J. Polym. Sci. Part B Polym. Phys..

[3] Jeon O., Bouhadir K.H., Mansour J.M., Alsberg E. (2009). Photocrosslinked alginate hydrogels with tunable biodegradation rates and mechanical properties. Biomaterials.

[4] Ning L., Chen X. (2017). A brief review of extrusion-based tissue scaffold bio-printing. Biotechnol. J..

[5] Correia Carreira S., Begum R., Perriman A.W. (2019). 3D Bioprinting: The Emergence of Programmable Biodesign. Adv. Healthc. Mater..

[6] Crivello J.V., Reichmanis E. (2014). Photopolymer Materials and Processes for Advanced Technologies. Chem. Mater..

[7] Pereira R.F., Bártolo P.J. (2015). 3D Photo-Fabrication for Tissue Engineering and Drug Delivery. Engineering.

[8] Lin C.-C., Ki C.S., Shih H. (2015). Thiol-norbornene photo-click hydrogels for tissue engineering applications. J. Appl. Polym. Sci..

[9] OECD (2018). Test No. 442D: In Vitro Skin Sensitisation.

[10] OECD (2018). Test No. 442E: In Vitro Skin Sensitisation.

[11] Strickland J., Zang Q., Paris M., Lehmann D.M., Allen D., Choksi N., Matheson J., Jacobs A., Casey W., Kleinstreuer N. (2017). Multivariate models for prediction of human skin sensitization hazard. J. Appl. Toxicol..

[12] Sabnis A., Rahimi M., Chapman C., Nguyen K.T. (2009). Cytocompatibility studies of an in situ photopolymerized thermoresponsive hydrogel nanoparticle system using human aortic smooth muscle cells. J. Biomed. Mater. Res. A.

[13] Leonhardt S., Klare M., Scheer M., Fischer T., Cordes B., Eblenkamp M. (2016). Biocompatibility of photopolymers for additive manufacturing. Curr. Dir. Biomed. Eng..

[14] Premi S., Wallisch S., Mano C.M., Weiner A.B., Bacchiocchi A., Wakamatsu K., Bechara E.J., Halaban R., Douki T., Brash D.E. (2015). Photochemistry. Chemiexcitation of melanin derivatives induces DNA photoproducts long after UV exposure. Science.

[15] Moody C.S., Hassan H.M. (1982). Mutagenicity of oxygen free radicals. Proc. Natl. Acad. Sci. USA.

[16] Rookmaaker M.B., van Gerven H.A.J.M., Goldschmeding R., Boer W.H. (2012). Solid renal tumours of collecting duct origin in patients on chronic lithium therapy. Clin. Kidney J..

[17] Trepiccione F., Pisitkun T., Hoffert J.D., Poulsen S.B., Capasso G., Nielsen S., Knepper M.A., Fenton R.A., Christensen B.M. (2014). Early targets of lithium in rat kidney inner medullary collecting duct include p38 and ERK1/2. Kidney Int..

[18] Roque A., Herédia V., Ramalho M., de Campos R., Ferreira A., Azevedo R., Semelka R. (2012). MR findings of lithium-related kidney disease: Preliminary observations in four patients. Abdom. Imaging.

[19] Aiff H., Attman P.-O., Aurell M., Bendz H., Ramsauer B., Schön S., Svedlund J. (2015). Effects of 10 to 30 years of lithium treatment on kidney function. J. Psychopharmacol..

[20] Christensen B.M., Zuber A.M., Loffing J., Stehle J.-C., Deen P.M.T., Rossier B.C., Hummler E. (2011). αENaC-Mediated Lithium Absorption Promotes Nephrogenic Diabetes Insipidus. J. Am. Soc. Nephrol..

[21] Davis J., Desmond M., Berk M. (2018). Lithium and nephrotoxicity: Unravelling the complex pathophysiological threads of the lightest metal. Nephrology (Carlton).

[22] Hiatt M.J., Matsell D.G., Little M.H. (2016). Chapter 25-Plasticity within the Collecting Ducts: What Role Does This Play in Response to Injury?. Kidney Development, Disease, Repair and Regeneration.

[23] Efrati S., Averbukh M., Berman S., Feldman L., Dishy V., Kachko L., Weissgarten J., Golik A., Averbukh Z. (2005). N-Acetylcysteine ameliorates lithium-induced renal failure in rats. Nephrol. Dial. Transpl..

[24] Benedikt S., Wang J., Markovic M., Moszner N., Dietliker K., Ovsianikov A., Grützmacher H., Liska R. (2016). Highly efficient water-soluble visible light photoinitiators. J. Polym. Sci. Part A Polym. Chem..

[25] Korbmacher C., Segal A.S., Fejes-Toth G., Giebisch G., Boulpaep E.L. (1993). Whole-cell currents in single and confluent M-1 mouse cortical collecting duct cells. J. Gen. Physiol..

[26] Gillman N., Lloyd D., Bindra R., Ruan R., Zheng M. (2020). Surgical applications of intracorporal tissue adhesive agents: Current evidence and future development. Expert Rev. Med. Devices.

[27] Sun A., He X., Li L., Li T., Liu Q., Zhou X., Ji X., Li W., Qian Z. (2020). An injectable photopolymerized hydrogel with antimicrobial and biocompatible properties for infected skin regeneration. NPG Asia Mater..

[28] Duchi S., Onofrillo C., O’Connell C.D., Blanchard R., Augustine C., Quigley A.F., Kapsa R.M.I., Pivonka P., Wallace G., Di Bella C. (2017). Handheld Co-Axial Bioprinting: Application to in situ surgical cartilage repair. Sci. Rep..

[29] OECD (1997). Test No. 471: Bacterial Reverse Mutation Test.

[30] Fairbanks B.D., Schwartz M.P., Bowman C.N., Anseth K.S. (2009). Photoinitiated polymerization of PEG-diacrylate with lithium phenyl-2,4,6-trimethylbenzoylphosphinate: Polymerization rate and cytocompatibility. Biomaterials.

[31] Lim K.S., Schon B.S., Mekhileri N.V., Brown G.C.J., Chia C.M., Prabakar S., Hooper G.J., Woodfield T.B.F. (2016). New Visible-Light Photoinitiating System for Improved Print Fidelity in Gelatin-Based Bioinks. ACS Biomater. Sci. Eng..

[32] Monteiro N., Thrivikraman G., Athirasala A., Tahayeri A., França C.M., Ferracane J.L., Bertassoni L.E. (2018). Photopolymerization of cell-laden gelatin methacryloyl hydrogels using a dental curing light for regenerative dentistry. Dent. Mater..

[33] Lee T.Y., Guymon C.A., Jönsson E.S., Hoyle C.E. (2004). The effect of monomer structure on oxygen inhibition of (meth)acrylates photopolymerization. Polymer.

[34] López de Arbina L., Gugliotta L.M., Barandiaran M.J., Asua J. (1998). Effect of oxygen on emulsion polymerisation kinetics: A study by reaction calorimetry. Polymer.

[35] Godar D.E., Gurunathan C., Ilev I. (2019). 3D Bioprinting with UVA1 Radiation and Photoinitiator Irgacure 2959: Can the ASTM Standard L929 Cells Predict Human Stem Cell Cytotoxicity?. Photochem. Photobiol..

[36] Ding H., Illsley N.P., Chang R.C. (2019). 3D Bioprinted GelMA Based Models for the Study of Trophoblast Cell Invasion. Sci. Rep..

[37] Lee C., O’Connell C.D., Onofrillo C., Choong P.F.M., Di Bella C., Duchi S. (2020). Human articular cartilage repair: Sources and detection of cytotoxicity and genotoxicity in photo-crosslinkable hydrogel bioscaffolds. Stem Cells Transl. Med..

[38] Agar N.S., Halliday G.M., Barnetson R.S., Ananthaswamy H.N., Wheeler M., Jones A.M. (2004). The basal layer in human squamous tumors harbors more UVA than UVB fingerprint mutations: A role for UVA in human skin carcinogenesis. Proc. Natl. Acad. Sci. USA.

[39] Erden A., Karagöz H., Başak M., Karahan S., Cetinkaya A., Avci D., Bugǧday I. (2013). Lithium intoxication and nephrogenic diabetes insipidus: A case report and review of literature. Int. J. Gen. Med..

[40] de Groot T., Alsady M., Jaklofsky M., Otte-Höller I., Baumgarten R., Giles R.H., Deen P.M.T. (2014). Lithium causes G2 arrest of renal principal cells. J. Am. Soc. Nephrol..

[41] Datta S., Das A., Chowdhury A.R., Datta P. (2019). Bioink formulations to ameliorate bioprinting-induced loss of cellular viability. Biointerphases.

[42] Stockley J.H., Evans K., Matthey M., Volbracht K., Agathou S., Mukanowa J., Burrone J., Káradóttir R.T. (2017). Surpassing light-induced cell damage in vitro with novel cell culture media. Sci. Rep..

[43] Fedorovich N.E., Oudshoorn M.H., van Geemen D., Hennink W.E., Alblas J., Dhert W.J.A. (2009). The effect of photopolymerization on stem cells embedded in hydrogels. Biomaterials.

[44] Utesch D., Splittgerber J. (1996). Bacterial photomutagenicity testing: Distinction between direct, enzyme-mediated and light-induced events. Mutat. Res./Environ. Mutagen. Relat. Subj..

[45] Wang L., Yan J., Fu P.P., Parekh K.A., Yu H. (2003). Photomutagenicity of cosmetic ingredient chemicals azulene and guaiazulene. Mutat. Res./Fundam. Mol. Mech. Mutagenes..

[46] Williams R.V., DeMarini D.M., Stankowski L.F., Escobar P.A., Zeiger E., Howe J., Elespuru R., Cross K.P. (2019). Are all bacterial strains required by OECD mutagenicity test guideline TG471 needed?. Mutat. Res./Fundam. Mol. Mech. Mutagenes..

[47] Zeiger E. (2019). The test that changed the world: The Ames test and the regulation of chemicals. Mutat. Res./Genet. Toxicol. Environ. Mutagenes..

[48] Cimino M.C. (2006). Comparative overview of current international strategies and guidelines for genetic toxicology testing for regulatory purposes. Environ. Mol. Mutagenes..

[49] Lilienblum W., Rapporteur, Scientific Committee on Consumer Safety (SCCS) (2014). Opinion on the Safety of Trimethylbenzoyl-Diphenylphosphineoxide (TPO) in Cosmetic Products, 27 March 2014, SCCS/1528/14.

[50] Manojlovic D., Dramićanin M.D., Miletic V., Mitić-Ćulafić D., Jovanović B., Nikolić B. (2017). Cytotoxicity and genotoxicity of a low-shrinkage monomer and monoacylphosphine oxide photoinitiator: Comparative analyses of individual toxicity and combination effects in mixtures. Dent. Mater..

